# Spatial Noise in Coupling Strength and Natural Frequency within a Pacemaker Network; Consequences for Development of Intestinal Motor Patterns According to a Weakly Coupled Phase Oscillator Model

**DOI:** 10.3389/fnins.2016.00019

**Published:** 2016-02-04

**Authors:** Sean P. Parsons, Jan D. Huizinga

**Affiliations:** Department of Medicine, Farncombe Family Digestive Health Institute, McMaster UniversityHamilton, ON, Canada

**Keywords:** interstitial cells of Cajal, weakly coupled oscillator, noise, gap junction

## Abstract

Pacemaker activities generated by networks of interstitial cells of Cajal (ICC), in conjunction with the enteric nervous system, orchestrate most motor patterns in the gastrointestinal tract. It was our objective to understand the role of network features of ICC associated with the myenteric plexus (ICC-MP) in the shaping of motor patterns of the small intestine. To that end, a model of weakly coupled oscillators (oscillators influence each other's phase but not amplitude) was created with most parameters derived from experimental data. The ICC network is a uniform two dimensional network coupled by gap junctions. All ICC generate pacemaker (slow wave) activity with a frequency gradient in mice from 50/min at the proximal end of the intestine to 40/min at the distal end. Key features of motor patterns, directly related to the underlying pacemaker activity, are frequency steps and dislocations. These were accurately mimicked by reduction of coupling strength at a point in the chain of oscillators. When coupling strength was expressed as a product of gap junction density and conductance, and gap junction density was varied randomly along the chain (i.e., spatial noise) with a long-tailed distribution, plateau steps occurred at pointsof low density. As gap junction conductance was decreased, the number of plateaus increased, mimicking the effect of the gap junction inhibitor carbenoxolone. When spatial noise was added to the natural interval gradient, as gap junction conductance decreased, the number of plateaus increased as before but in addition the phase waves frequently changed direction of apparent propagation, again mimicking the effect of carbenoxolone. In summary, key features of the motor patterns that are governed by pacemaker activity may be a direct consequence of biological noise, specifically spatial noise in gap junction coupling and pacemaker frequency.

## Introduction

A few consistent observations have been made of slow waves, or the contractions they drive, in the small intestine. Commonly they travel in an aboral direction; their frequency decreases aborally; this frequency gradient often consists of a series of flat plateaus bounded by abrupt steps; clamping the intestine, so as to block conduction, decreases the frequency aboral to the clamp (e.g., Alvarez, 1914; Hasselbrack and Thomas, 1961; Christensen et al., 1966; Diamant and Bortoff, 1969; Szurszewski et al., 1970). The first of these has an obvious and critical function, to encourage food to travel aborally rather than orally along the gut. Early on it was speculated that the other three are aspects of the mechanism underlying the first (Alvarez, 1928). This mechanism is now understood as coupled oscillator theory (Pavlidis, 1973; Winfree, 1980).

Slow waves are generated in the small intestine by a network of interstitial cells of Cajal (ICC-MP) that covers the entire length and circumference of the intestine at the level of the myenteric plexus (Sanders et al., 2014) with a density of around 1000 cells per mm^2^ (Mei et al., 2009). Each ICC-MP can generate a rhythmic depolarization in isolation (Koh et al., 1998; Thomsen et al., 1998). In coupled oscillator theory each element of a network oscillates in isolation but this oscillation is influenced by the oscillations of its neighboring, coupled oscillators. In general two coupled oscillators will tend to minimize both their phase difference, a process called synchronization, and their frequency difference, called entrainment. Synchronization was first observed by Huygens in the seventeenth century between pendulums (Bennett et al., 2002) and explains how networks of oscillators can generate waves. The wave does not propagate, instead it is a coordinated phase difference across the oscillator network, a “phase wave.” When all the oscillators have the same frequency, the phase difference can go to zero (complete synchronization) and the phase wave will have an infinite velocity. If there is a gradient in frequency across the network then the higher frequency oscillators will always be ahead of the phase of the lower frequency oscillators and so the phase wave will appear to propagate from the high to the low frequency part of the network. The velocity of the phase wave will vary, as the phase difference between different frequency oscillators necessarily changes with time. The frequency difference can be minimized by entrainment. Here a distinction must be made between the oscillator's natural frequency, the frequency at which it oscillates in isolation, and the frequency it oscillates at in a network. Entrainment is when an oscillator with a higher natural frequency, “pulls” up the oscillation frequency of an oscillator with a lower natural frequency, toward its own frequency. How much depends on the strength of coupling and difference in natural frequency between the oscillators. In a chain of oscillators with a natural frequency gradient, entrainment pulls a length of the chain to the same frequency, a frequency plateau. A series of plateaus can result, with intervening steps where the frequency difference is too great for entrainment. At a frequency step one of every few phase waves will terminate as a “dislocation.” For instance if plateau A and its neighbor B have wave intervals of 1.5 and 1.4 s, respectively, then every 15th wave in A will terminate at the step between A and B (Parsons and Huizinga, 2015a).

**Figure 1:**
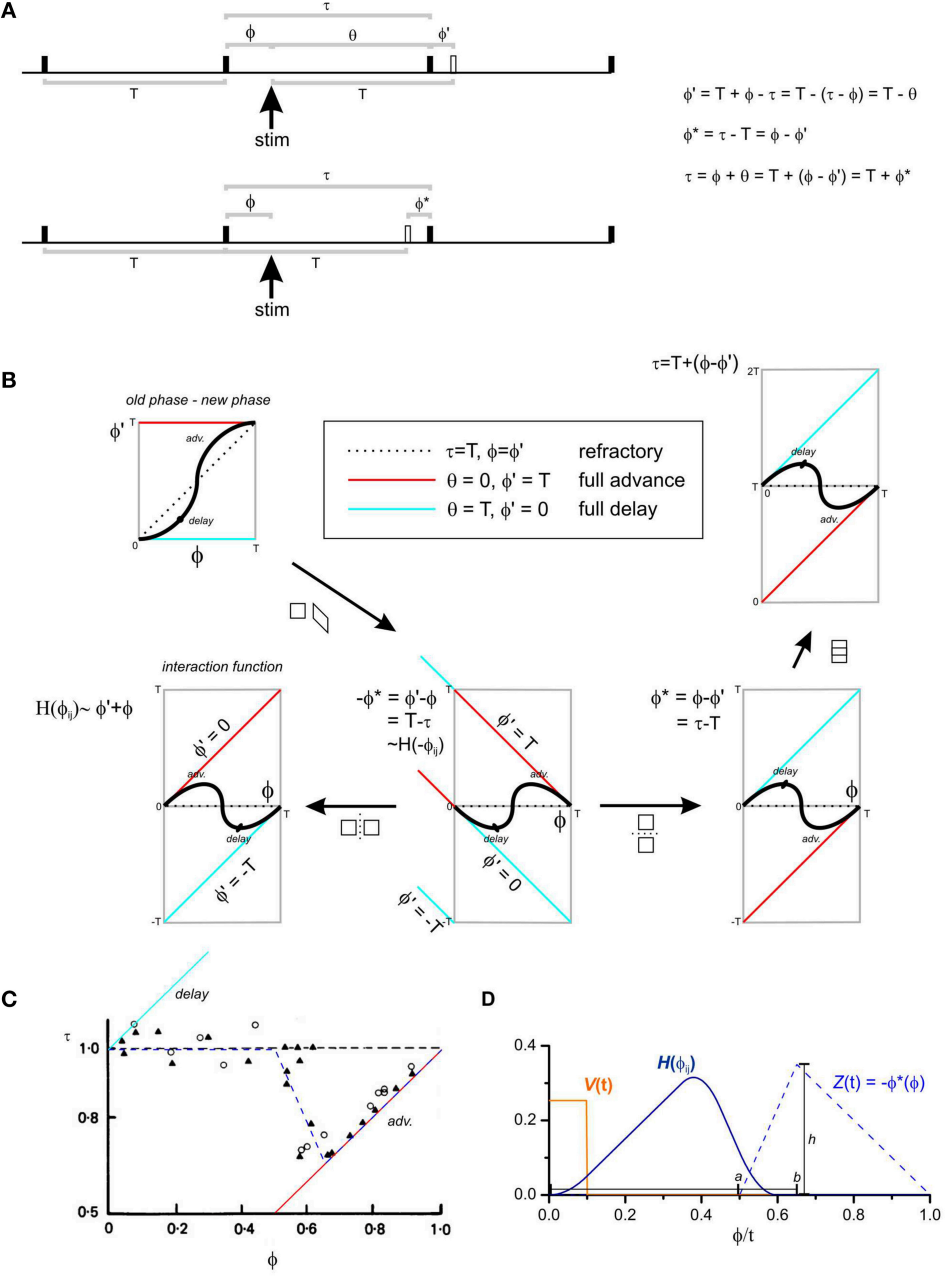
**Phase response curves and the interaction function**. **(A)** An oscillator is represented by a horizontal time line and “fires” at each solid black uptick. The firing time could be defined as the upstroke of the slow wave. The oscillator has a natural period of *T*. *T* and all other variables can be defined either in units of radians (*T* = 2π), seconds (*T* = some value) or normalized to *T* (*T* = 1). A stimulus is given ϕ after a fire and the next fire is τ after this fire. Further measurements can be made, grouped according to two standpoints: (1) *Upper time line*, the oscillator “should have” fired *T* after the stimulus (*hollow uptick*). ϕ′ is the phase difference between when the oscillator did fire and when it “should have.” If ϕ′ = T, the oscillator fired immediately upon stimulation—the phase of the oscillator was fully advanced (*box*, center of **B**). Conversely if ϕ′ = 0, the oscillator fired at the time it “should have”—the phase of the oscillator was fully delayed (*box*, center of **B**). If ϕ′ = ϕ, then the stimulus had no effect on the firing of the oscillator—it was refractory. Another measurement used is the cophase (θ) the time between the stimulus and the first fire. (2) *Lower time line*, the oscillator “should have” fired *T* after it's last fire (*hollow uptick*). ϕ^*^ is the phase difference between when the oscillator did fire and when it “should have.” **(B)** There are a number of conventions for the plotting of phase response curves. The abscissa is always the phase of the stimulus (ϕ), but the ordinate can be ϕ′, −ϕ^*^, ϕ^*^, or τ (anticlockwise from left). ϕ′(ϕ) is known as an “old phase—new phase” phase response curve (Winfree, 1980). All the phase response curves can be related by simple geometric transforms (arrows). The interaction function, *H*(ϕ) is a reversed and smoothed version of the infinitesimal phase response curve −ϕ^*^(ϕ) (Equation 5). **(C)** τ(ϕ) with *T*-normalized units for slow waves recorded from the rabbit small intestine, modified from Figure 3A of Cheung and Daniel (1980). Triangles and circles are circular and longitudinal muscle responses, respectively, to 5 ms, 80 V pulses. The slow waves are refractory until halfway through their cycle at which point they phase advance, reaching full phase advance (responding immediately to a stimulus) about 7/10 of the way through the cycle. The blue dashed line is the infinitesimal phase response curve used for our model (Equation 6). **(D)** Model infinitesimal phase response curve *Z*(*t*), impulse *V*(*t*) and interaction function *H*(ϕ_*ij*_). *a, b*, and *h* are the parameters used to define *Z*(*t*) (Equation 6).

Coupled oscillator theory coalesced from many different components. Of these, van der Pol's work on relaxation oscillators was particularly important. In a relaxation oscillation the amplitude increases gradually for part of its cycle (relaxes) and then abruptly increases before decreasing again. Van der Pol discovered entrainment in electrical circuits that acted as relaxation oscillators and described a simple differential equation that could model this (van der Pol, 1926; van der Pol and van der Mark, 1927). He also noted that the rhythmicity of the heart had the properties of a relaxation oscillator (van de Pol, 1940). In 1961 Fitzhugh showed that the Hodgkin Huxley equations could be simplified to a second order differential equation that contains the van de Pol equation as a limiting case, now called the Fitzhugh-Nagumo or Bonhoeffer-van der Pol equation (Fitzhugh, 1961). In the same year Bortoff introduced coupled oscillator theory to explain the properties of slow waves in the small intestine (Bortoff, 1961). A decade later Diamant showed that a chain of Fitzhugh-Nagumo oscillators could reproduce the frequency plateaus of the small intestine (Diamant et al., 1970). This was followed by many studies on chains of coupled relaxation oscillators by gastrointestinal physiologists (Brown et al., 1971, 1975; Sarna et al., 1971, 1972; Specht and Bortoff, 1972; Robertson-Dunn and Linkens, 1974; Akwari et al., 1975; Linkens, 1977; Linkens and Datardina, 1977; Publicover and Sanders, 1989; Daniel et al., 1994; Aliev et al., 2000; Gizzi et al., 2010). In 1967 Winfree published his insight that when coupling between oscillators is “weak,” in the sense that oscillators only influence each other's phase and not their amplitude (no oscillator deviates significantly from its limit cycle), then the dynamics of any such oscillator can be described by a single differential equation of the oscillator's phase (Winfree, 1967, 1980). This idea was developed over the next two decades into what is now known as the theory of weakly coupled oscillators (Schwemmer and Lewis, 2012). It provides a practical means to “phase reduce” any model, biophysical or otherwise, of two or more coupled oscillators to a single first order differential equation (Equation 1). This equation has only two variables, natural frequency and coupling strength, and a function, the interaction function. Significantly for the physiologist all but one of these can be determined from experimental data. Natural frequency can be measured from the uncoupled oscillation frequency or estimated from the coupled gradient of oscillation frequency. For the interaction function, a stimulus, such as a current pulse for electrical oscillators, is given at different phases of the oscillator's cycle and the change in phase induced by this stimulus measured (Figure 1A). This gives a phase response curve which can be used to derive the interaction function (Figures 1B,C).

In a previous paper we studied contraction waves in the murine small intestine using the technique of diameter mapping (Parsons and Huizinga, 2015a). We observed frequency plateaus and dislocations, and showed how these were affected when coupling was reduced by the gap junction blocker carbenoxolone. Our aim here was to model this data using a chain of weakly coupled phase oscillators. All but one parameter of the model were fixed using experimental physiological data. We discovered that spatial noise in coupling strength and natural frequency of the pacemaker network play a decisive role in the orchestration of intestinal motor patterns. Part of this work has been published previously in abstract form (Parsons and Huizinga, 2015b).

## Materials and methods

### Experiments and analysis

All procedures were approved and carried out in accordance with regulations of the Animal Ethics Board of McMaster University. Organ bath video recordings of the murine small intestine were made and diameter maps (DMaps) created, as described previously (Parsons and Huizinga, 2015a). A DMap is an image where the *x* axis corresponds to time, the *y* axis to distance along the length of the intestine (in all maps shown, top to bottom corresponds to proximal to distal). The image intensity at {*x, y*} corresponds to the intestine diameter at that time and distance, with black corresponding to contracted and white, relaxed.

The tone of the small intestine usually varies along its length and so there are variations in DMap intensity along its *y*/distance axis. To enhance the contrast of contractions and aid comparison with DMaps simulated by the model, intensity was equalized across the *y* axis. For each point on the *y* axis the mean intensity was calculated across *x* and this was subtracted from all pixels at that point.

To measure the frequency of waves in DMaps, experimental or modeled, autocorrelation was calculated for each point on the *y*/distance axis for lags up to 10 s. This gave a {lag, *y*} image with intensity on a blue-white-red scale, where blue corresponds to negative correlation, white near to zero and red positive correlation. Autocorrelation is always +1 at zero lag, then proceeds to oscillate within the bounds of −1 and +1. The first positive peak, after zero-lag, corresponds to the correlation between every consecutive wave, the second peak to the correlation between every other wave, etc. The lag of each peak is the interval between waves.

### Model

Different authors in the weakly coupled oscillator literature use different terminology, sign conventions and symbols for the same equations. In the following we stay as close as possible to the usage of Schwemmer and Lewis (2012). The model consisted of a chain of *n* coupled phase oscillators. The rate of change of phase (θ) of the *i*th oscillator is,

(1) $$\begin{array}{rcl} \frac{d\theta_{i}}{dt} & = & \omega_{i}+\frac{1}{2}\underset{j}{\sum }k_{ij}H\left(\phi_{ij}\right)\\ i & = & 1\ldots .n\\ \omega_{i} & = & 2\pi ∕\zeta_{i}\\ j & = & i-1,i+1\\ \phi_{ij} & = & \left(\theta_{j}-\theta_{i}\right)\;\mathrm{mod}\;2\pi \end{array}$$

where ω is the natural frequency (radians/s); ζ is the natural interval (s); *k* is the coupling strength (unitless); *H* is the interaction function (radians/s). *n* was 200, large enough to give a fairly fine grained simulation and small enough for a fast numerical solution.

The model was solved in MATLAB (MathWorks, Natick, MA) using the ode45 Runge-Kutta method. It was found that the solution was sensitive to rounding errors in θ as θ became larger. To avoid these errors the model was solved in consecutive blocks of 100/max(ω) seconds. The 2π modulo of the last phases of one block were used as the initial phases for the next block (the initial phases for the first block were random). In this way θ never exceeded much beyond 100. With a Intel Core i5-2300 (2.8 GHz) processor it typically took 3–4 s for a 250 s simulation of 200 oscillators. Solutions were returned at an interval of 33 ms, the video sample interval for the experimental DMaps. DMaps (with a *y*/length axis of *n* pixels) were created from the solution by converting phases to amplitudes by sine function.

The natural interval (ζ) was normally distributed with standard deviation σ_ζ_ about a linear gradient along the chain from ζ_low_ to ζ_high_,

(2) $$\begin{array}{l} \zeta_{i}=\zeta_{low}+i\left(\zeta_{high}-\zeta_{low}\right)∕n+\sigma_{\zeta }U \end{array}$$

where *U* is a standard normal variate. Based on data from the murine small intestine, ζ_low_ was 1.2 s and ζ_high_ was 1.5 s (Figure 4A of Parsons and Huizinga, 2015a). σ_ζ_ was varied (see Section Results).

**Figure 2:**
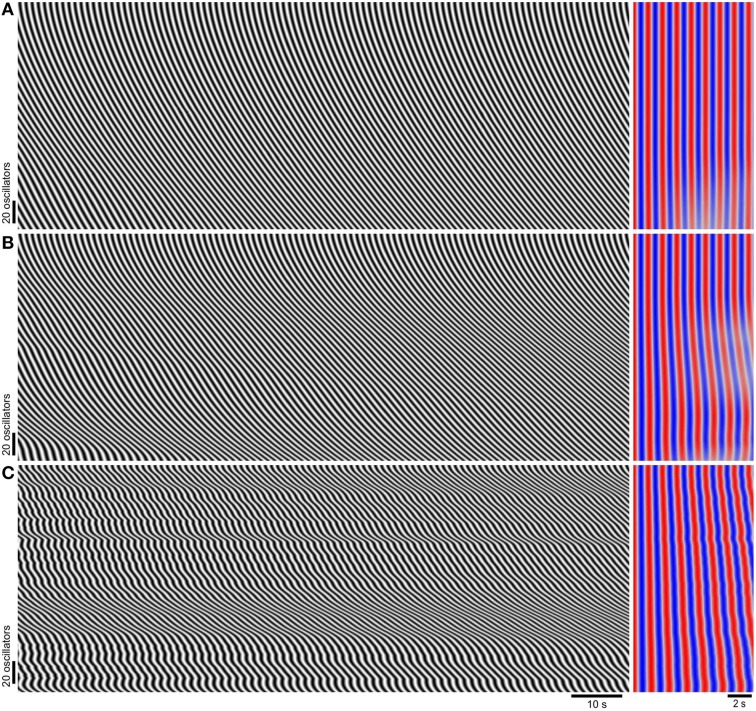
**Phase wave patterns and frequency as coupling strength (*k*) is varied**. Model diameter maps (*left*), and their autocorrelation (*right*). The top of the maps correspond to the proximal (higher natural frequency) end. Time scales are indicated below **(C)**. Autocorrelation lag runs from left to right and red is positive correlation while blue is negative. After zero lag the first peak in correlation (first red band) corresponds to correlation between every consecutive oscillation, the second peak (second red band) corresponds to correlation between every other oscillation and so on (see Section Materials and Methods). The model has a noiseless frequency gradient (ζ_*low*_ = 1.2 s, ζ_*high*_ = 1.5 s, σ_ζ_ = 0 s) and uniform coupling strength. **(A)** *k* = 3. **(B)** *k* = 1. **(C)** *k* = 0.1.

**Figure 3:**
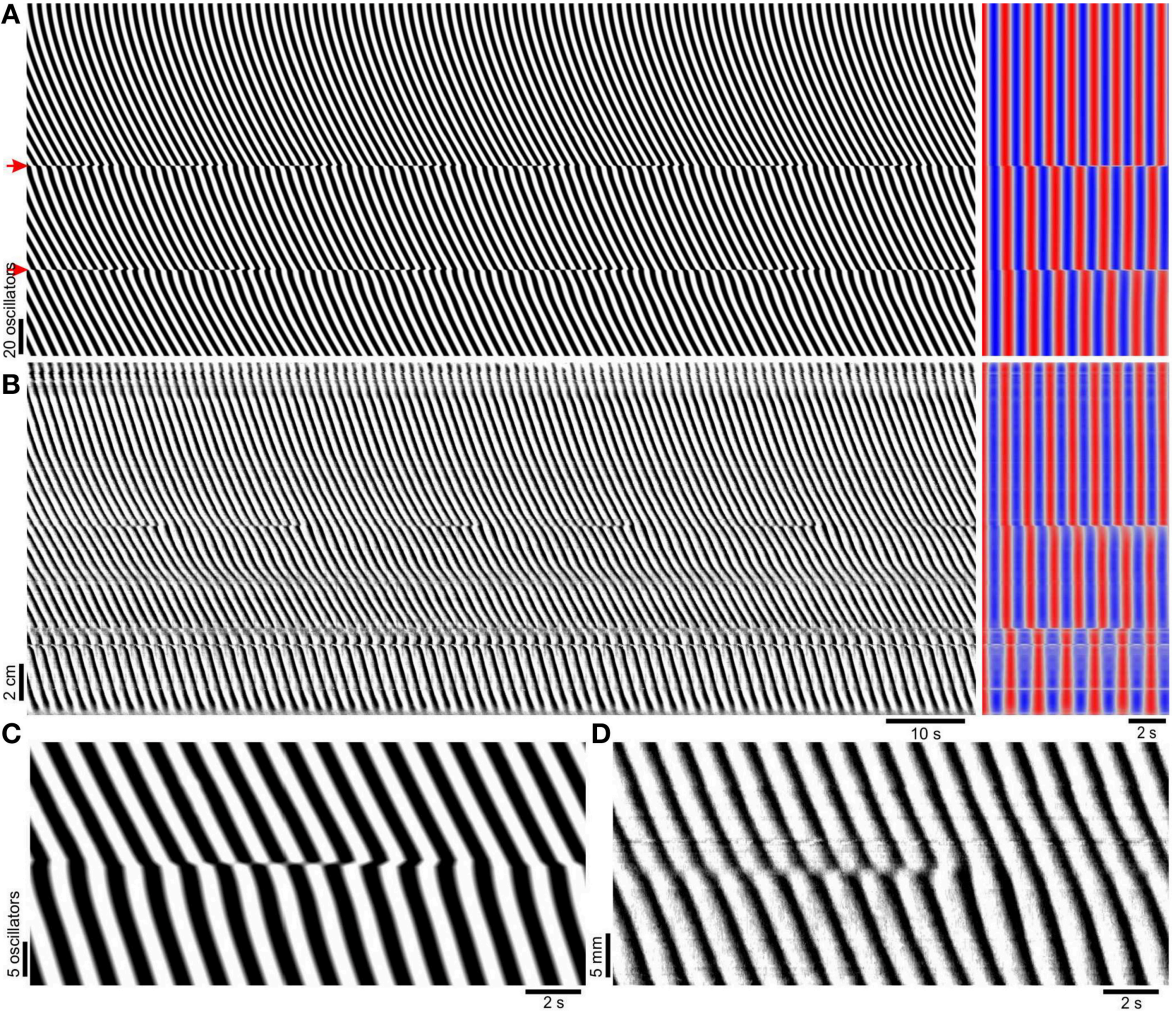
**Induction of frequency steps by localized decoupling**. **(A)** Model diameter map (*left*), and its autocorrelation (*right*). The top of the map corresponds to the proximal (higher frequency) end. Time scales are indicated below **(B)**. The model has the same parameters as in Figure 2A, but with coupling strength reduced by 90% (*k* = 0.3) at the points marked with red arrows. At the points of reduced coupling there are clear steps in frequency, corresponding to positions of dislocations in the DMap. **(B)** Experimental diameter map of murine small intestine (*left*) in the presence of 0.5 mM lidocaine to block enteric nervous system activity and its autocorrelation (*right*). The top of the map corresponds to the proximal end. **(C)** A dislocation from the model DMap in **(A)**. **(D)** A dislocation from the small intestine DMap in **(B)**.

**Figure 4:**
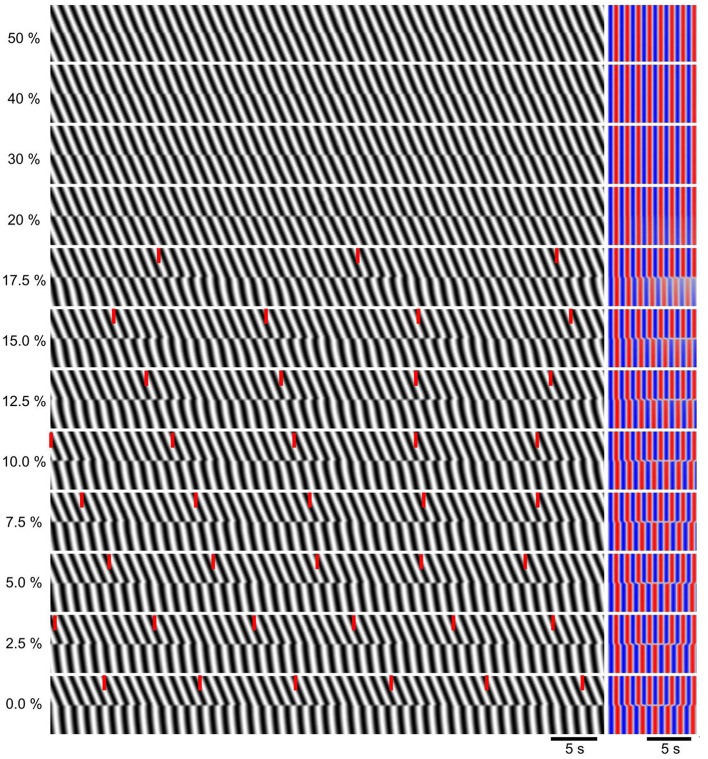
**Dependence of dislocations on strength of localized decoupling**. All 200 oscillators had the same coupling strength (*k* = 3), except between oscillators 100 and 101 where the coupling was reduced to a percentage of this, indicated at left. *Left*, DMaps showing the middle 20 oscillators. Red ticks indicate times of wave drops (dislocations). *Right*, corresponding autocorrelations.

Coupling strengths (*k*) were symmetrical (*k*_*ij*_ = *k*_*ji*_). In the simplest model, *k* was the same value for all oscillators except at selected points (Figures 2–4). In a more complex model (**Figures 6**, **7**), *k* was the product of a gap junction conductance (*g*_junc_) and gap junction density (*d*_junc_). A decrease in *g*_junc_ modeled gap junction blockers. *d*_junc_ had a reversed Lévy distribution restricted between 0 and 1,

(3) $$\begin{array}{rcl} k_{ij} & = & k_{ji}=g_{junc}d_{junc}\\ d_{\text{junc}} & = & 1-X \end{array}$$

where *X* was a random variate from the Lévy stable distribution (Weron and Weron, 2005) with the characteristic function,

(4) $$\begin{array}{l} \varphi \left(t\right)=\exp\left[-\sigma^{\alpha }|t{|}^{\alpha }\left(1-i\beta \text{sgn}\left(t\right)\tan\left(\pi \alpha ∕2\right)\right)+it\mu \right] \end{array}$$

with characteristic exponent α = 0.5, skewness β = 0.5, scale s = 0.02, and location μ = 0. Random variates were generated with the code of Veillette (2008) until a variate was in the range (0, 1).

The interaction function (*H*) is periodic over the period *T* = 2π radians. It is proportional to the one sided convolution over *T*, of the infinitesimal phase response curve of the oscillator (*Z*) and the impulse of the neighboring oscillator (*V*),

(5) $$\begin{array}{l} \;H\left(\phi_{ij}\right)=\frac{1}{T}\int_{0}^{T}Z(t)\left[V(t+\phi_{ij})-V(t)\right]dt\\ \;=\frac{1}{T}\int_{0}^{T}Z(t)V(t+\phi_{ij})dt-\frac{1}{T}\int_{0}^{T}Z(t)V(t)dt\\ \;=\frac{1}{T}\int_{0}^{T}Z(t)V(t+\phi_{ij})dt-C\\ H\left(-\phi_{ij}\right)=\frac{1}{T}\int_{0}^{T}Z(t)V(t-\phi_{ij})dt-C \end{array}$$

Thus, if the impulse is a square wave, the interaction function is a reversed and smoothed version of the infinitesimal phase response curve, with the impulse as the “boxcar” smoothing kernel (Figure 1D). When the impulse is a delta function, *H*(−ϕ_*ij*_) ~ *Z*(ϕ_*ij*_).

We based the model's infinitesimal phase response curve on the experimental phase response curve of slow waves in the rabbit small intestine determined by Cheung and Daniel (1980) (Figure 1C). Ideally the infinitesimal phase response curve represents the phase response to an infinitely short stimulus (a delta function). The stimulus used by Cheung and Daniel was 5 ms, 1/740 of the 3.7 s average cycle length, so we felt brief enough for approximation. The infinitesimal phase response curve was,

(6) $$\begin{array}{l} Z(t)=\{\begin{array}{cc} 0 & t<a\\ h\frac{t-a}{b-a} & a<t<b\\ -h\frac{x-2\pi }{2\pi -b} & t>b \end{array}\\ \;a=\pi \\ \;b=1.3\pi \\ \;h=2\pi -b \end{array}$$

where *a* is the length of the cycle when the oscillator is refractory to a stimulus and; *b* is the point in the cycle beyond which the oscillator is fully phase advanced by a stimulus (i.e., responds to a stimulus by immediate “firing” or fully synchronizes with the stimulating oscillator) (Figure 1D).

Physiologically the impulse is the part of the ICC depolarization that can effect a neighboring, coupled ICC (it doesn't have to correspond to the whole slow wave, just up to where there is no more phase change induced). This was modeled as a simple square wave of width *w*, as a fraction of the complete cycle,

(7) $$\begin{array}{l} V(t)=\{\begin{array}{cc} {\left(2\pi w\right)}^{-1} & t\leq 2\pi w\\ 0 & t>2\pi w \end{array}\\ \;w=0.1 \end{array}$$

Z(*t*) and *V*(*t*) were one side convolved to create a “look up table” of *H*(ϕ_*ij*_) at discrete values of ϕ. This was passed to the ODE evaluation function and *H* for a particular value of ϕ was found from it by linear interpolation.

## Results

### Frequency steps and localized decoupling

The parameters of the model were determined as much as possible by experimental data. The natural frequency gradient was estimated from the observed contraction intervals at either end of the mouse intestine, 1.2–1.5 s, proximal to distal (Parsons and Huizinga, 2015a). The interaction function was calculated from the phase response curve of slow waves in the rabbit small intestine (Cheung and Daniel, 1980) and a square impulse representing the depolarising phase of the slow wave. This left one free parameter, the coupling strength, *k*.

When *k* was varied over a wide range, phase waves propagated distally along the length of the chain but there were no frequency steps or dislocations (Figure 2). At higher coupling strengths the whole chain was entrained to the same frequency (Figure 2A). The lack of frequency steps was not due to coupling being so strong as to result in entrainment to a single oscillator (the proximal, highest frequency oscillator). At lower coupling strengths entrainment weakened but instead of steps and plateaus forming, frequency varied smoothly, eventually following the natural frequency gradient (Figures 2B,C). Also the phase wave velocity increasingly oscillated as coupling was reduced. Smooth variations in wave frequency or velocity of this magnitude were not seen in the mouse small intestine (Parsons and Huizinga, 2015a and Figure 3B). In previous coupled oscillator models frequency steps occurred spontaneously given a large enough frequency gradient (Diamant et al., 1970; Brown et al., 1971, 1975; Sarna et al., 1971, 1972; Specht and Bortoff, 1972; Robertson-Dunn and Linkens, 1974; Akwari et al., 1975; Linkens, 1977; Linkens and Datardina, 1977; Publicover and Sanders, 1989; Kopell et al., 1990; Daniel et al., 1994). It appeared that the physiological frequency gradient in the mouse was not large enough to explain frequency steps. Instead we made the novel hypothesis that steps in the mouse result from localized decoupling between ICC at the frequency step. Reducing coupling between two oscillators would allow a distal (lower natural frequency) oscillator to escape entrainment by its proximal neighbor and thus allow a plateau step to form. Indeed when this was done by reducing *k* by 90% at two points, two frequency steps (three plateaus) formed and dislocations occurred rhythmically at these points (Figure 3A). This pattern was very similar to that seen in diameter maps of the whole mouse small intestine *in vitro* (Figure 3B). Wave velocity decreased distally across each plateau in both the model and intestine (Figures 3A,B) as phase lag increased due to the larger difference between the oscillator's natural and entrained frequency (Somers and Kopell, 1995). Dislocations were also very similar between the intestine and model (Figures 3C,D).

As *k* was incrementally reduced between the middle two oscillators of the chain, there was no distinguishable change until *k* was reduced to 40% of the control value of three, when there was an instantaneous change in phase at the point of reduction, a “phase slip.” (Figure 4). As *k* was reduced further the magnitude (phase difference) of this slip increased, until at 20% the waves proximal and distal to the reduction point were anti phase. At 17.5% a frequency step and wave drops were induced. With further reduction down to 0%, the frequency of wave drops increased as the magnitude of the step increased.

### Spatial noise in coupling strength

In our previous study of the small intestine, frequency steps were very stable in position. Inhibition of gap junctions with carbenoxolone induced new steps but the old steps were conserved (**Figures 6B,E** in Parsons and Huizinga, 2015a). This suggests to us that step positions reflect underlying structural discontinuities rather than being the result of a physiochemical dynamic equilibrium. The model suggests that these discontinuities are a reduction in coupling strength (Figure 3), either in the density of gap junctions or equivalently the density of ICC or cellular connections between ICC. It is possible that discontinuities occur at well spaced points against a uniform background as modeled above (Figure 3A), perhaps through a targeted developmental mechanism. Or perhaps more likely the coupling strength could vary randomly along the length of the intestine, at certain points falling below the threshold to give steps. In other words the coupling strength would be spatially noisy.

**Figure 5:**
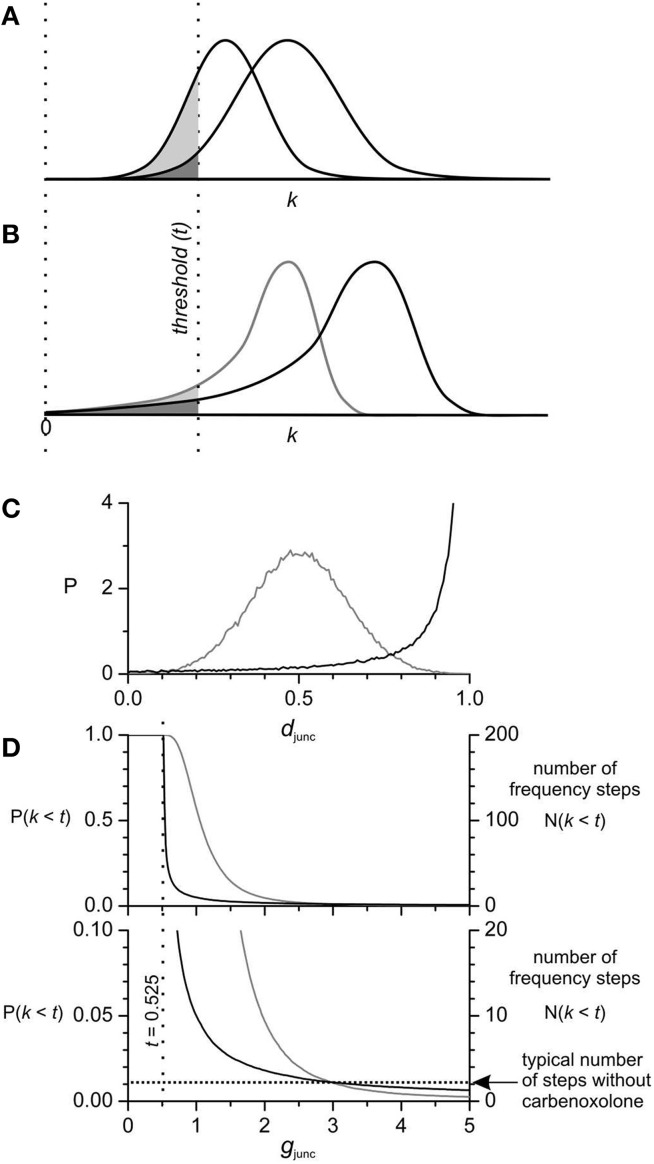
**Coupling strength (*k*) distribution and the number of frequency steps**. The number of steps is proportional to the area of the *k* distribution left of the threshold (*t*) for inducing steps. As the distribution of *k* is contracted P(*k*) → P(*k*/*a*) or shifted left P(*k*) → P(*k* − *a*), the area left of threshold increases and so does the number of steps. Starting with normal **(A)** and long left tailed **(B)** distributions with *equal* area left of threshold (*dark shading*), when each is contracted by the same amount the area left of threshold (*light shading*) is much greater in the normal distribution. The same relative effect occurs with shift. Thus, the parameters of a normal distribution have to be much more finely tuned to obtain a particular number of steps. This is important when considering coupling strength in the small intestine because the number of steps does not vary widely between intestines, nor does the number of steps increase exponentially with reduction of coupling by carbenoxolone. In our model *k* is the product of a gap junction density (*d*_junc_), which varies between oscillators, and a gap junction conductance (*g*_junc_), a single value for all oscillators. Thus, the distribution of *k* is an expansion/contraction of the distribution of *d*_junc_, according to the value of *g*_junc_. **(C)** The Lévy-stable distribution of *d*_junc_ used in our model (Section Materials and Methods) has a long left tail (*black line*) in comparison to a normal distribution (*gray line*). Both distributions were calculated numerically (*n* = 10^5^). Based on the data in Figure 3 the threshold for step induction is *k* = 0.525 (17.5% of *g*_junc_ = 3). **(D)** Lévy-stable and Gaussian *d*_junc_ distributions were adjusted so that the corresponding *k* distributions had equal areas left of threshold [P(*k* < 0.525)] at *g*_junc_ = 3, so starting with the same number of steps [N(*k* < *t*)] (two, a typical number without carbenoxolone). As *g*_junc_ is decreased from three and the distribution of *k* is contracted, N(*k* < *t*) rises much faster for the normal distribution (*gray line*) than for the Lévy-stable distribution (*black line*). Both reach P(*k* < *t*) = 1, as *g*_junc_ goes to *t* (the whole of the *k* distribution is to the left of *t*). Similarly as *g*_junc_ is increased above three, N(*k* < *t*) decreases further for the normal distribution, but only decreases a little for the Lévy-stable distribution. Again the conclusion is that *g*_junc_ has to much more finely tuned with a normal distribution of *d*_junc_ then with a long-left tailed distribution.

**Figure 6:**
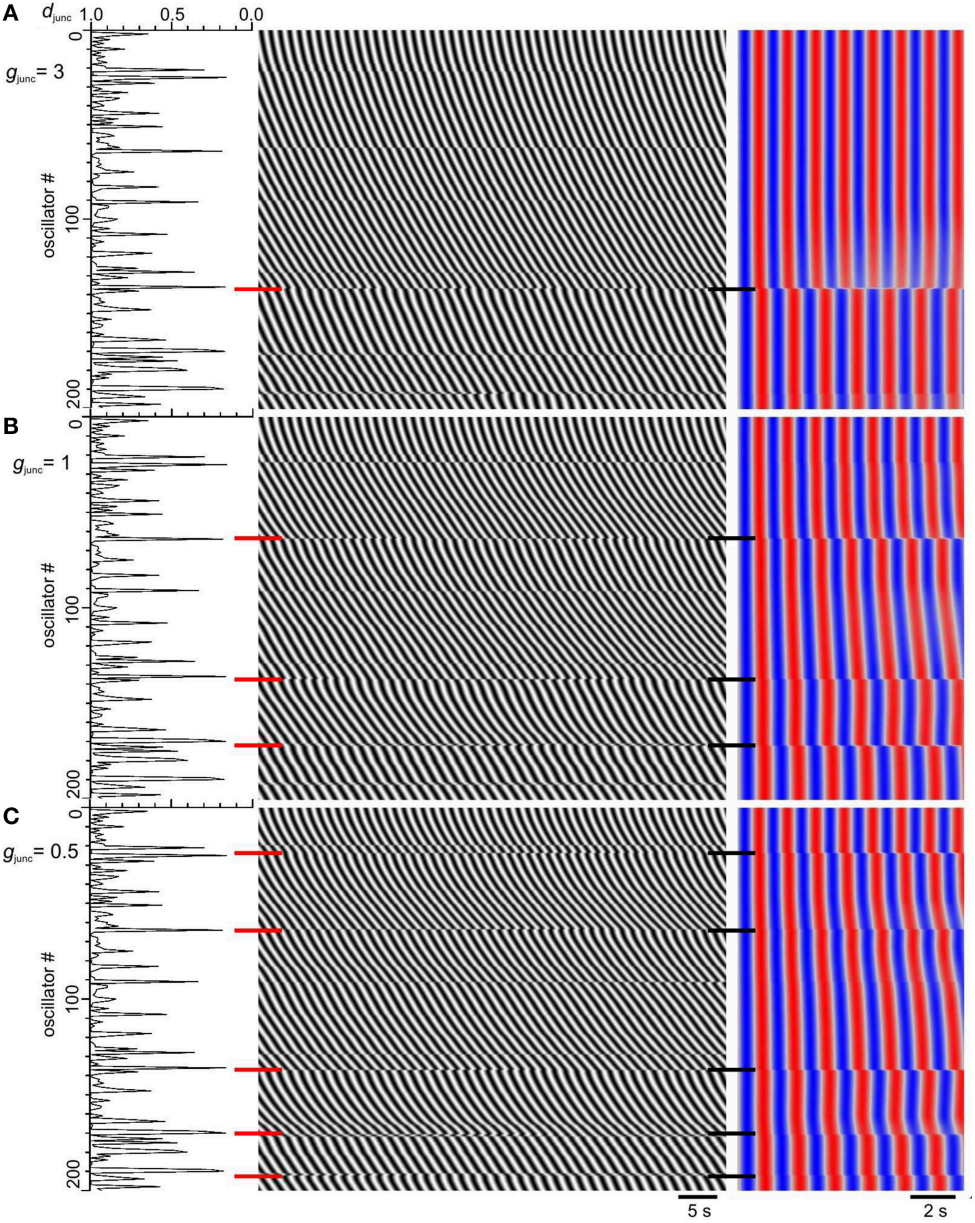
**Increase in frequency steps as gap junction conductance is decreased**. Model *d*_junc_ (*left*), DMaps (*middle*), and autocorrelation (*right*). The top of the maps correspond to the proximal (higher frequency) end. Gap junction density (*d*_junc_) had a long tailed distribution and gap junction conductance (*g*_junc_) was either 3 **(A)**, 1 **(B)**, or 0.5 **(C)**. *Red and black horizontal bars*, frequency step positions.

If coupling strength is spatially noisy, its distribution must be known to model it. Given the low number of steps under basal conditions and that their number only increases 2–3 times with carbenoxolone (Figure 6 in Parsons and Huizinga, 2015a) we hypothesized that the distribution of coupling strengths should have a long left tail (Figure 5). That way if the threshold for step formation is on the tail, the vast majority of coupling strengths will be above threshold and the relative position of the tail and threshold can vary widely without greatly changing the fraction of strengths below threshold, the number of steps (Figure 5B). We put these considerations into a model for the coupling strength (Equation 3) (Figure 5). *k* was the product of a gap junction conductance (*g*_junc_) and a gap junction density (*d*_junc_). *d*_junc_ was varied randomly along the chain between 0 and 1 according to a Lévy distribution, a long tailed distribution. *g*_junc_ was a single value that was decreased to model inhibition of gap junction conductance. Steps occurred at points of low *d*_junc_ (Figure 6A). As *g*_junc_ was decreased six fold, the number of steps/plateaus increased from ~1–2 to 5–10 (Figures 6B,C), in line with the increase seen with carbenoxolone in the small intestine (Figure 6 in Parsons and Huizinga, 2015a).

### Spatial noise in natural frequency

**Figure 7:**
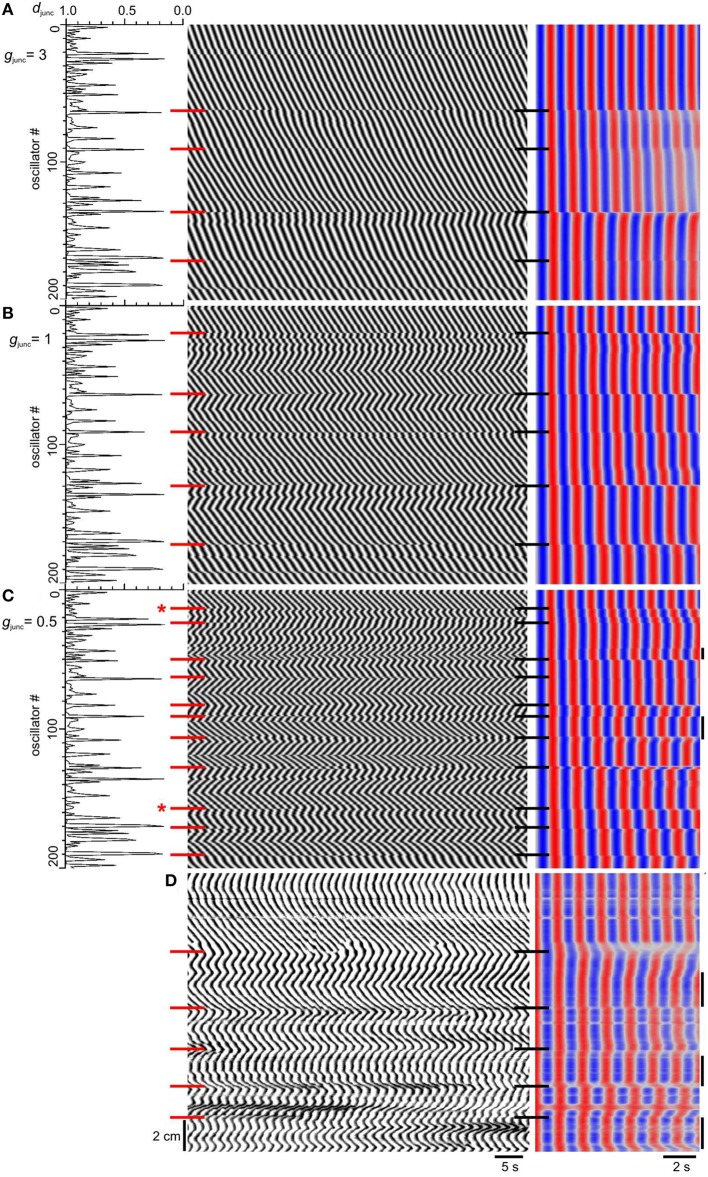
**Effect of natural frequency spatial noise on emergence of frequency steps**. **(A–C)** Model *d*_*junc*_ (*left*), DMaps (*middle*) and autocorrelation (*right*). The top of the maps correspond to the proximal (higher frequency) end. The natural intervals were distributed normally about their gradient (1.2–1.5 s) with a standard deviation of 30 ms. Gap junction density (*d*_junc_) had a long tailed distribution and gap junction conductance (*g*_junc_) was either 3 **(A)**, 1 **(B)** or 0.5 **(C)**. *Red and black horizontal bars*, frequency step positions. Bars with asterisk indicate where frequency steps are not coincident with dips in *d*_*junc*_. High frequency islands are indicated by vertical bars at the right of the autocorrelation. **(D)** DMap and autocorrelation of murine small intestine in the presence of 40 μM carbenoxolone. The top of the map corresponds to the proximal end.

Carbenoxolone produced a zigzagging reversal of wave direction (Figure 7D here and Figure 6A in Parsons and Huizinga, 2015a), but this was not observed when we decreased *g*_junc_ (Figure 6). In the presence of carbenoxolone, “high frequency islands” (plateaus with higher frequency than both their distal and proximal neighbors) were seen (Parsons and Huizinga, 2015a) and this gave us a hypothesis as to the development of the zigzagging waves. We hypothesized that natural frequency is spatially noisy, but the magnitude of this noise is normally insignificant compared to the natural frequency change over the span of a plateau. As plateaus get shorter with gap junction inhibition, their natural frequency span also gets smaller, to within the range of the natural frequency noise. Therefore, the chances increase that a local spike in natural frequency will be the highest frequency within its plateau, entraining the slow wave frequency within its plateau, and be higher than any natural frequency in the neighboring proximal plateau, resulting in a high frequency island. Spatial noise in natural frequency would also explain the zigzagging seen with carbenoxolone as local gradients in natural frequency coupled with local variations in coupling strength drive the phase wave in different directions. To test our hypothesis, the natural intervals were varied randomly with a normal distribution of 30 ms standard deviation, about the 1.2–1.5 s proximal to distal gradient. The magnitude of this variation is comparable to the temporal variation of slow wave frequency measured experimentally. It gives a coefficient of variation (*c*_v_ = standard deviation/mean) of 0.022 (0.03/1.35). In the dog small intestine (Table 1 of Szurszewski et al., 1970) slow wave frequency for a single animal was 14.8 ± 0.08 S.E. min^−1^ (measured as slow waves over a minute period), *n* = 17 and so *c*_v_ = 0.08√17∕14.8 = 0.022. As *g*_junc_ was decreased the number of plateaus increased as before, but also the phase waves zigzagged and high frequency islands developed (Figures 7A–C). Waves formed a V across each plateau (Figure 7C) exactly like the small intestine in the presence of carbenoxolone (Figure 7D). Also the frequency between steps was often not uniform, varying smoothly in the manner of an oscillating slope rather than a plateau (Figure 7C) and again this was seen in the small intestine (Figure 7D). In some cases frequency steps did not correspond with minima in junction density (Figure 7C).

## Discussion

Random variation, spatial, or temporal noise, is a natural property of any biological system. The role of temporal noise in slow wave generation has been recognized in the form of stochastic miniature depolarizations called unitary events or spontaneous transient depolarizations (Hashitani et al., 1996; Edwards et al., 1999; Hirst and Edwards, 2001). Here we show that spatial noise in key parameters of the pacemaker network play a decisive role in the orchestration of intestinal motor patterns. The evidence was provided by using experimental data to constrain the few parameters of a chain of weakly coupled phase oscillators and introducing spatial noise.

There are many mechanisms through which a particular distribution of coupling strength could be achieved. Long tailed distributions of connectivity (node degree) in mathematical networks (graphs) can be produced by targeted removal or addition of connections (edges) at nodes that already have few or more connections, respectively (Barabasi and Albert, 1999; Barrat et al., 2008), i.e., positive feedback. Targeted addition is called preferential attachment. A long left tailed distribution of coupling strength could result if connections were removed between already poorly connected ICC (low *d*_junc_). In the brain an excess of neural connections are formed during embryonic development and this is followed postnatally by targeted removal of synapses, dendrites, and neurons themselves, according to each neuron's activity (Hua and Smith, 2004) and thus dependent on the number of connections. This was first observed by Cajal (Schuldiner and Yaron, 2015). He called it “process resorption” and it is now called “pruning.” Gao and colleagues have shown that pruning occurs in the myenteric ICC network (Gao et al., 2013a, 2014). ICC density reaches a peak 2 weeks after birth and after this the distance between ICC increases. Though it is not known whether this pruning is targeted, the fact that it occurs 2 weeks after birth, by which time ICC generate slow waves, suggests the possibility of activity-dependent preferential detachment. It is also possible that gap junction density spatial noise could be independent of a variation in ICC density, ICC having varying gap junction expression levels. There is no data on this. Or gap junction density may not itself be noisy but rather the gap junction conductance. Conductance is determined by the unitary conductance and the open probability and these are controlled by pH and connexin phosphorylation (Nielsen et al., 2012) which could remain stable over minutes.

Imtiaz et al. (2006) applied weakly coupled oscillator theory to slow waves starting with a biophysical model of a single oscillator based on a decade of experimental dissection of the slow wave by van Helden, Hirst, Kito and Suzuki (Hirst and Ward, 2003; van Helden et al., 2010 for review). Each oscillator has two pools of calcium, the cytosol and an intracellular store. Release from the store to the cytosol is through inositol trisphosphate (IP_3_) gated channels, with the synthesis of IP_3_ modulated by membrane potential. A rise in cytosolic calcium depolarises membrane potential (such as through calcium activated chloride channels) and causes further store calcium release (i.e., calcium induced calcium release). In this way oscillations are generated by feedback between calcium, IP_3_ and membrane potential. Imtiaz et al. determined the −ϕ^*^(ϕ) phase response curve for a single oscillator stimulated with a depolarising pulse (Figures 2, 14 of Imtiaz et al., 2006) and this was almost identical to the experimental phase response curve of Cheung and Daniel (1980), used here. The model cell was refractory for the first half of its cycle, dipped to a small phase delay near T/2, after which the cell rapidly switched to full phase advance. When two oscillators were coupled they synchronized (Imtiaz et al., 2006) and when coupling was lowered in the middle of a chain of oscillators, the oscillators at either side would desynchronize (van Helden and Imtiaz, 2003). This modeled an ingenious experiment where they recorded slow waves from two ends of a strip of gastric muscle and then applied glycyrrhetinic acid (an analog of carbenoxolone) or 2-APB (an IP_3_R inhibitor) to the intervening portion as a perfused stream (van Helden and Imtiaz, 2003). In the model the sensitivity of stores to IP_3_ was varied randomly across the array of cells with a normal distribution. As this sensitivity largely determined natural frequency it parallels, and could be a biological basis for, the spatial noise of the natural frequency in our model.

Imtiaz et al. also analyzed the interaction function by phase reducing their biophysical model (Imtiaz et al., 2006). The rate of change of the phase difference (ϕ) between two symmetrically coupled, equal frequency oscillators as a function of ϕ is

(8) $$\begin{array}{l} \frac{d\phi }{dt}=\frac{d\theta_{i}}{dt}-\frac{d\theta_{j}}{dt};\phi =\theta_{i}-\theta_{j}\\ \;=k\left[H\left(\theta_{j}-\theta_{i}\right)-H\left(\theta_{i}-\theta_{j}\right)\right]\\ \;=k\left[H\left(-\phi \right)-H\left(\phi \right)\right]\\ \;=-kH_{odd}\left(\phi \right);H_{odd}=H\left(\phi \right)-H\left(-\phi \right)\\ \;=kG\left(\phi \right);G\left(\phi \right)=-H_{odd}\left(\phi \right)\;\left(8\right) \end{array}$$

The odd part of the interaction function (*H*_*odd*_), or the equivalent growth function (*G*), give the equilibrium values of ϕ, where *G* or *H*_*odd*_ is zero with a slope < 0 (*G*) or > 0 (*H*_*odd*_). Thus, one can determine not just that the oscillators can synchronize, but also whether they might stably oscillate out of phase in “phase locked” states. Imtiaz et al. found that as the basal rate of IP_3_ synthesis increased, *H*_*odd*_ presented a series of phase locked states (Imtiaz et al., 2006). We did not present *G*(ϕ) for our interaction function because we were primarily concerned with a qualitative analysis and the extra complications of our model (spatial noise and gradients over multiple oscillators) would confound a simple interpretation of *G*(ϕ). In our model, neighboring oscillators are always incrementally out of phase (the phase waves have a finite velocity) because of the natural frequency gradient. *G*(ϕ) is not responsible for this, but if it were there would have to be one equilibrium point very close to ϕ = 0. Stable phase differences of larger magnitude that might reflect *G*(ϕ) equilibrium points at significant distant from ϕ = 0, were seen at phase slips, but these were at points of decoupling, independent of *G*(ϕ). The magnitude of the phase slip increased incrementally, again independently of any change in *G*(ϕ), as the transition between complete entrainment (synchrony) and induction of frequency steps (Figure 4).

Ermentrout and Kopell, “partially motivated by certain phenomena observed in mammalian small intestine” (Ermentrout and Kopell, 1984), modeled chains of coupled phase oscillators with a natural frequency gradient (Ermentrout and Kopell, 1984, 1991; Kopell and Ermentrout, 1986, 1990; Kopell et al., 1990). As with earlier papers on chains of coupled relaxation oscillators (see Section Introduction) frequency plateaus arose without decoupling. In these cases the frequency gradient was often in the region of 50% to obtain 2–3 plateaus vs. 20% in our model. This greater frequency difference was probably enough on its own to break entrainment. Somers and Kopell (1995) gave a proof that when a phase oscillator chain is divided into domains that are out of synchrony with each other (such as with frequency plateaus or decoupling) then waves will propagate in both directions from the center of each domain. This phenomenon, which they called fractured waves, can be clearly seen in both our model and the experimental data (Figure 7). For each plateau, with boundaries determined by decoupling, waves spread in a V from the center of the plateau. However, we only saw this in the model when we introduced the natural frequency spatial noise. Somers and Kopell make no mention of this in their proof, however they do say that in addition to being independent domains, the boundary oscillators at neighboring domains must be out of phase. This might depend on increasing their natural frequency difference with noise.

A waxing and waning of contraction amplitude occurs at frequency steps in the small intestine (Diamant and Bortoff, 1969; Suzuki et al., 1986; Parsons and Huizinga, 2015a). This likely results from superposition of waves at either side of the step, an effect well characterized in acoustics (Discussion of Parsons and Huizinga, 2015a). A weakly coupled oscillator model can have little to say about this interpretation as it does not include amplitude as a variable. Oscillator phase can be converted to amplitude by a fixed function (as done here by sine function) but as each oscillator must pass through the full phase range, the amplitude range during each cycle will also always be the same. Waxing waning of contraction amplitude can occur in the absence of frequency steps through an unrelated mechanism, modulation of slow wave amplitude by a lower frequency pacemaker activity (“phase-amplitude modulation”) (Huizinga et al., 2014).

Arrays of cellular automata have been used to model slow wave propagation in the small intestine (Lammers et al., 2011; Gao et al., 2013b). Lammers et al. (2011) found that when an increasing percentage of cells were made inactive, propagation first slowed and then began to fail. The percentage of active cells would be analogous to coupling strength in our model and though we did not measure velocity changes in the model, propagation velocity does decrease with decoupling by carbenoxolone in the small intestine (Parsons and Huizinga, 2015a).

Weakly coupled oscillator theory is extremely powerful as it can distill into a single equation, biophysical models, via phase reduction, and experimental data and classical physiological concepts such as refractory period, via the phase response curve. Thus, one can tie together biophysics, mathematical analysis, experiment, and modeling to reveal connections that might not otherwise be apparent and better allowing one to feed the other. We have demonstrated a novel connection between spatial variation or noise and the physiological and experimental phenomena of frequency step stability, V-waves and high frequency islands, a connection that would not have been in any way obvious or trivial without the guide of the theory. This connection suggests further experimental, biophysical and mathematical investigations.

## Author contributions

SP designed and executed the experiments, and wrote the manuscript. JH supervised and contributed to manuscript writing.

### Conflict of interest statement

The authors declare that the research was conducted in the absence of any commercial or financial relationships that could be construed as a potential conflict of interest.
